# Complementary and alternative therapies for non-alcoholic fatty liver disease: A Bayesian network meta-analysis protocol

**DOI:** 10.1097/MD.0000000000024432

**Published:** 2021-01-29

**Authors:** Tiefeng Zhang, Duan Han, Tianqi Zhang, Cai Jing, Jianguang Sun

**Affiliations:** a First College of Clinical Medicine, Shandong University of Traditional Chinese Medicine; b Second Affiliated Hospital of Shandong University of Traditional Chinese Medicine; c Affiliated Hospital of Shandong University of Traditional Chinese Medicine, Jinan, Shandong Province, China.

**Keywords:** Bayesian, complementary and alternative therapies, network meta-analysis, non-alcoholic fatty liver disease, protocols

## Abstract

**Background:**

Non-alcoholic fatty liver disease (NAFLD) has become a global pandemic, and its incidence is increasing year by year. At present, there are no definite curative drugs for the treatment of NAFLD in modern medicine. Surprisingly, complementary and alternative therapies play an important role and have special advantages. In this study, we will adopt Bayesian network meta-analysis (NMA) to evaluate the efficiency and safety of complementary therapy and alternative therapies for NAFLD.

**Methods:**

We will collect randomized controlled trials (RCTs) related to the treatment of NAFLD in PubMed, Cochrane Library, CNKI, and other databases. Two reviewers will screen the literature and extract data in line with the inclusion and exclusion criteria, and then assess the risk of bias according to Cochrane risk of bias assessment tool. The Bayesian NMA will be performed by Stata16.0 and WinBUGS1.4.3.

**Results:**

Our study will compare and rank the efficacy and safety of diverse complementary and alternative therapies for NAFLD.

**Conclusion:**

This study can provide credible evidence for the efficacy and safety of complementary therapies and alternative therapies in the treatment of NAFLD. We expect to assist clinicians and patients to choose the optimal therapeutic regimen.

**Protocol registration number:**

INPLASY2020120136.

## 1 Introduction

As the epidemics of obesity and type 2 diabetes mellitus increase worldwide, the prevalence of non-alcoholic fatty liver disease (NAFLD) is increasing proportionately. The latest epidemiological studies show that the number of patients with NAFLD accounts for 25% of the global population and its incidence varies from region to region and social conditions, ranging from 22.1% to 28.6%.^[1]^ A growing number of studies have manifested that NAFLD is a multi-system disease, which not only affects the structure and function of liver, but also increases the incidence rate of type 2 diabetes, cardiovascular diseases, cerebrovascular diseases, and chronic kidney disease.^[2]^ The pathogenesis of NAFLD is mainly related to the imbalance of lipid metabolism, insulin resistance, inflammation activation, endoplasmic reticulum stress, and intestinal microbial imbalance.^[3,4]^ NAFLD can develop from simple steatosis to non-alcoholic steatohepatitis (NASH), fibrosis, cirrhosis, hepatocellular carcinoma and necrosis.^[5]^

The current consensus on NAFLD treatment is not only to control the NAFLD disease itself, but also to manage related metabolic comorbidities such as obesity, type 2 diabetes, hyperlipidemia, and insulin resistance.^[6]^ In addition, the overall mortality rate of NAFLD is much higher than that of the general population, and the prognosis is worse once they develop to liver cirrhosis and liver cancer. Therefore, standardized, diversified and personalized treatment of NAFLD is particularly significant. At present, the first-line recommendation on NAFLD is lifestyle intervention, which mainly includes the adjustment of the diet structure and appropriate exercise.^[7]^ Besides, recognized therapeutic drugs mainly involve insulin sensitizers, lipid-lowering drugs, antioxidants, liver protection drugs, probiotics, etc.^[8–10]^ However, long-term medication can also cause liver injury, gastrointestinal reactions, allergic reactions, edema, and other adverse effect. Moreover, withdrawal reactions are prone to occur after stopping the medication. When lifestyle interventions and drug therapy are ineffective, bariatric surgery can be an effective intervention for obese patients, which can improve the insulin resistance and steady state of adipose tissue and reduce the risk of liver fibrosis.^[11]^ Nevertheless, the long-term benefits of weight loss therapy need further study. Patients who meet the surgical indications can undergo liver transplantation, and there is also the risk of organ rejection and recurrence after liver transplantation. In a word, the current treatment strategy for NAFLD is mainly aimed at lifestyle intervention and controlling the risk of metabolism and complications. Although some progress has been achieved in treatment in recent years, the efficacy of related treatment options is still controversial.

Thus, it is extremely urgent to find an effective and safe interventional therapy. Recently, it is well recognized that complementary and alternative therapies play an essential role in NAFLD. Generally speaking, these therapies for NAFLD mainly contain dietary supplements, massage, acupuncture, acupoint injection, Chinese herbal medicine, psychotherapy, exercise therapy, etc.^[12]^ The latest research has demonstrated that the mechanism of action of traditional Chinese medicine (TCM) intervention is mainly reflected in regulating lipid metabolism, increasing insulin sensitivity, inhibiting oxidative stress, improving the intestinal barrier, and improving inflammation.^[13]^ Besides, acupuncture and moxibustion also have a certain effect on the treatment of NAFLD. For example, Fenglong point can reduce the expression of SREBP-1c in NAFLD rats, improve endoplasmic reticulum stress, and regulate abnormal lipid metabolism.^[14]^ In addition, Vitamin E plays an important role in improving alanine aminotransferase (ALT), aspartate aminotransferase (AST), NAFLD activity score (NAS), liver fibrosis degree.^[15,16]^ Moreover, studies have shown that exercise training reduces intrahepatic fat and FFAs while increasing cardiorespiratory fitness.^[17]^

With the advancement of modern medicine, interventions for NAFLD are gradually diversified and there are more options to choose. Faced with multiple interventions, traditional meta-analysis limited by pairwise comparisons can no longer offer effective methodological support for choosing the optimal interventions. Consequently, the presentation of network meta-analysis (NMA) makes it possible to compare the difference among multiple interventions. The most outstanding characteristics of NMA is that it can be analyzed quantitatively and compared after summarizing different intervention for similar diseases. Hence, in this study, NMA will be performed to find the appropriate complementary and alternative therapies for NAFLD.

## 2 Methods

### 2.1 Study registration

Our study has been registered on the International Platform of Registered Systematic Review and Meta-analysis Protocols (INPLASY). The registration number is: INPLASY2020120136 (URL = https://inplasy.com/inplasy-2020–12–0136/). Besides, we will perform the protocol according to the Preferred Reporting Items for Systematic Review and Meta-Analysis protocols specification for reporting strictly.^[18]^

### 2.2 Inclusion criteria

#### 2.2.1 Type of study

This study will contain RCTs related to the treatment of NAFLD. The sample size of both the treatment group and the control group should be at least 30. The language will be restricted to Chinese or English.

#### 2.2.2 Participants

Patients who have been diagnosed as non-alcoholic fatty liver disease (NAFLD) will be included. There are no restrictions on gender and age. Patients combined with other chronic liver diseases such as alcoholic liver disease, various viral hepatitis, autoimmune liver disease, drug-induced hepatitis will be excluded. Additionally, liver cirrhosis, liver cancer, severe liver disease requiring liver transplantation, and other complicated and severe patients are also not included.

#### 2.2.3 Interventions

NAFLD patients in the treatment group will be given complementary and alternative therapies, including dietary supplements, massage, acupuncture, acupoint injection, Chinese herbal medicine, psychotherapy, exercise therapy, etc. Different complementary and alternative therapies can be freely combined. What's more, they can be used based on the routine western medicine or independently.

#### 2.2.4 Comparisons

The control group will be given with conventional western medicine. There is no limitation on the dosage form.

#### 2.2.5 Outcomes

1. Primary outcomes: total effective rate, main blood lipid indexes (TC, TG), main liver function indexes (ALT, AST).
2. Secondary outcomes: TCM Syndrome Score Scale (TCMSSS), body mass index (BMI), fasting blood glucose, ultrasound imaging changes, and the incidence of adverse events, etc.

### 2.3 Exclusion criteria

1. Basic research on animal experiments and cell experiments.
2. Literature reviews, case reports, and experience summaries.
3. Patients with other chronic liver diseases such as alcoholic liver disease, various viral hepatitis, autoimmune liver disease, drug-induced hepatitis.
4. Patients with liver cirrhosis, liver cancer, severe liver disease requiring liver transplantation, and other complicated and severe disease.
5. Literature with incomplete data, poor quality, or published repeatedly.

### 2.4 Databases and search strategy

The search databases are as follows: PubMed, Cochrane Library, Cochrane Controlled Trial Center Registration, EMBASE, CNKI, Wanfang Database, VIP Database. We will collect all relevant RCTs about NAFLD. Retrieval time is from the database inception to November 2020. We will optimize the search strategy according to the characteristics of diverse databases. The search strategy will be in the form of medical subject headings (MeSH) and keywords. Furthermore, we will continue to follow up literature in the systematic review/meta-analysis. The search strategy of PubMed database is shown in Table 1.

**Table 1:** Details of the search strategy for PubMed.

No.	Search item
#1	non-alcoholic fatty liver disease[MeSH Terms]
#2	Non alcoholic Fatty Liver Disease [Title/Abstract] ORNAFLD [Title/Abstract]ORNonalcoholic Fatty Liver Disease [Title/Abstract] OR Fatty Liver, Nonalcoholic [Title/Abstract] OR Liver, Nonalcoholic Fatty [Title/Abstract] OR Nonalcoholic Fatty Liver [Title/Abstract] ORNonalcoholic Steatohepatitis[Title/Abstract] ORnon-alcoholic steatohepatitis [Title/Abstract]OR Steatohepatitides, Nonalcoholic [Title/Abstract] ORfatty liver[Title/Abstract]
#3	#1 OR #2
#4	Complementary Therapies [MeSH Terms]
#5	Complementary and alternative therapies [Title/Abstract] OR Alternative Medicine [Title/Abstract] OR Alternative Therapies [Title/Abstract] OR Complementary Medicine [Title/Abstract] OR Herbal therapy [Title/Abstract] OR Herb Therapy [Title/Abstract] OR Therapy, Alternative [Title/Abstract] OR Therapy, Complementary [Title/Abstract] OR Complementary Therapies [Title/Abstract] OR Therapies, Alternative [Title/ Abstract]
#6	#4 OR #5
#7	dietary supplement [Title/Abstract] OR Massage [Title/Abstract] OR Acupuncture [Title/Abstract]OR Chinese herbal medicines [Title/Abstract] OR Moxibustion [Title/Abstract] OR Acupoint injection [Title/Abstract] OR psychotherapy [Title/Abstract] OR exercise [Title/Abstract]
#8	#6 OR #7
#9	Randomized Controlled Trial [Publication Type] OR Controlled Clinical Trial [Publication Type] OR Randomized [Title/Abstract] OR randomly [Title/Abstract] OR random allocation [Title/Abstract]
#10	#3 AND #8 AND #9

### 2.5 Data extraction

Two researchers (Tiefeng Zhang and Duan Han) will respectively browse the title and abstract, sort out the literature in accordance with the inclusion and exclusion criteria, and then extract the data. For the qualified literature, we will search the full text to obtain more information. When 2 independent researchers encounter the discrepancy, they will discuss or ask a third-party reviewer (Jianguang Sun) for help to solve the problem. If the collected literature is incomplete, we will contact the corresponding author to supplement relevant information. According to the above retrieval strategy, we will retrieve all relevant literature and then import them into EndnoteX9. We will strictly follow the principles of PICO, and the information to be extracted mainly includes title, author, journal, year, the trial registration number, gender, age, sample size, intervention measures and outcome indicators, adverse reactions, etc.

### 2.6 Risk of bias analysis

Two review authors will evaluate the quality of RCTs via Cochrane risk of bias assessment tool, which contains 7 items and every item is classified into “high”, “unclear” and “low” levels.^[19]^ The 2 authors will exchange the results to determine whether they are consistent. In case of any objection, the third author shall participate in the discussion and decide the final result.

### 2.7 Assessment of heterogeneity

We will apply the Chi-Squared test and *P* value to qualitatively analyze the heterogeneity of each research result. When *I*^2^ < 50% and *P* > .10, there is no significant heterogeneity, we will adopt a fixed effects model. Otherwise, we will perform a random effects model. What's more, the heterogeneity caused by experimental design, duration of treatment, and other factors needs subgroup and sensitivity analysis. Besides, descriptive analysis will be adopted considering the indefinite sources of heterogeneity.

#### 2.7.1 Subgroup analysis

When *I*^2^ > 50% and *P* value <.1, there is obvious heterogeneity between the studies, therefore it is essential to analyze the reasons for that. We will conduct a subgroup analysis according to the source of heterogeneity. For instance, if it is due to the quality of methodology, subgroup analysis will be undertaken in the light of the quality. If it is due to the difference in design, we will analyze age, gender, treatment type, and course of disease.

#### 2.7.2 Sensitivity analysis

We will assess the sensitivity analysis via excluding literature one by one, so as to find out whether the literature has effect on heterogeneity. When the heterogeneity of research changes after excluding a literature, the literature may be the source of the heterogeneity, and we will further analyze the following factors, such as the difference in sample size, the reference standard of the outcomes, etc. Otherwise, the sensitivity is slight and the results are more credible.

### 2.8 Statistical analysis

#### 2.8.1 Pairwise meta-analysis

We will conduct a conventional paired meta-analysis for direct comparison results of the literature. Besides, continuous data are represented by the weighted mean difference (WMD), standardized mean difference (SMD) and 95% confidence interval (CI), while dichotomous data will be calculated by the risk ratio (OR) and 95%CI.

#### 2.8.2 NMA

We will use STATA 16.0 and WinBUGS1.4.3 for NMA to compare direct and indirect evidence.^[20]^ In WinBUGS 1.4.3 software, the Bayesian framework is simulated by Markov-chain-Monte-Carlo (MCMC).^[21]^ After statistical analysis, the potential scale reduction factor (PSRF) will be applicable to evaluate convergence. When the result comes closer to 1, the convergence and the conclusion are more credible. If the PRSF exceeds this range, we will continue to manually increase the number of iterations until the FRSF is within this range. In addition, we will regulate iterations and annealing times based on the actual situation. Numerical variables will be represented by SMD with 95%CI. Then, WinBUGS 1.4.3 software will be utilized to rank the effectiveness of different interventions. Our study will apply the subsurface cumulative ranking curve values to forecast and rank the treatment effect. The higher the percentage, the better the intervention effect will be. In addition, we will assess the consistency model of the principal outcomes and the probability ranking of the optimum treatment. If there is a closed loop, we will use the node splitting method to appraise the inconsistency between indirect comparison and direct comparison.

### 2.9 Publication bias

If meta-analysis contains more than 10 studies, a funnel chart will be applied to assess the publication bias. We can evaluate the influence of publication bias by observing its symmetry. If the funnel chart is symmetric, there is no significant publication bias. If not, there may be publication bias. However, publication bias is not the only reason for the asymmetry of the funnel chart. There are other sources, such as low-quality small sample trials, poor method design, insufficient analysis, and data authenticity.

### 2.10 Evaluation of evidence quality

We will use the GRADE method to evaluate the quality of evidence and strength of recommendations.^[22]^ Nowadays, GRADE is the most valuable tool for assessing the quality of NMA evidence, which can be divided into high, medium, low, and very low.

## 3 Discussion

NAFLD can not only cause chronic liver dysfunction, but also lead to various serious complications, such as cardiovascular diseases, chronic kidney disease, colorectal cancer and endocrine diseases.^[23]^ Currently, there is no specific drug for NAFLD in modern medicine, so complementary and alternative therapies are quite necessary.^[24]^ Studies have shown that these therapies have significant advantages in NAFLD, which can reduce liver cell damage, promote the lipid metabolism, and improve the degree of hepatic steatosis.^[25,26]^ However, it is not sufficient to draw a conclusive conclusion on the efficacy and safety of these therapies. For clinicians, how to choose the most appropriate combination of complementary and alternative therapies is still a problem. Therefore, this study uses NMA to explore the efficacy and safety of complementary and alternative therapies for NAFLD, and then offer reliable evidence support for clinicians to make the best options.

NMA is a method developed from traditional meta-analysis, which expands from the standard meta-analysis of 2 groups of trials to the simultaneous analysis and comparison of multiple diverse therapeutic factors. The greatest advantage of NMA is that it can quantitatively compare different interventions for treating similar diseases, and sort them according to the effect of a certified outcomes, so as to choose the optimal treatment. However, we must realize that NMA still has yet to be discussed in the methodology. First of all, the randomized controlled design is not rigorous enough and the standards are not clear enough, so the quality of the included literature is uneven. Secondly, our research is based on the meta-analysis of published RCTs, rather than the original data, so the results themselves are also biased. Third, although strict search and inclusion criteria have been stipulated, some literature may be omitted. Therefore, we wish to see more high-quality, prospective, multicenter RCTs in the future to obtain more reliable evidence-based medicine. Anyhow, this study will offer trustworthy results for the optimum complementary and alternative therapy, and provide powerful evidence for its significant benefits in the treatment of NAFLD.

## Author contributions

**Conceptualization:** Jianguang Sun.

**Formal analysis:** Tiefeng Zhang, Duan Han.

**Methodology:** Tianqi Zhang, Cai Jing.

**Project administration:** Tiefeng Zhang, Jianguang Sun.

**Software:** Duan Han, Cai Jing.

**Writing – original draft:** Tiefeng Zhang, Duan Han.

**Writing – review & editing:** Tiefeng Zhang, Tianqi Zhang, Jianguang Sun.

## Abbreviations

Abbreviations: GRADE = Grading of Recommendations Assessment, Development and Evaluation, MeSH = medical subject term, NAFLD = non-alcoholic fatty liver disease, RCTs = randomized controlled trials, TC = total cholesterol, TG = triglycerides.
